# Effects of Hydrothermal Pretreatment and Hydrochar Addition on the Performance of Pig Carcass Anaerobic Digestion

**DOI:** 10.3389/fmicb.2021.622235

**Published:** 2021-04-12

**Authors:** Jie Xu, Hongjian Lin, Kuichuan Sheng

**Affiliations:** ^1^School of City and Architecture Engineering, Zaozhuang University, Zaozhuang, China; ^2^College of Biosystems Engineering and Food Science, Zhejiang University, Hangzhou, China

**Keywords:** pig carcass, hydrothermal temperature, hydrochar addition, biogas production, ammonia inhibition

## Abstract

Proper disposal and utilization of dead pig carcasses are problems of public concern. The combination of hydrothermal pretreatment (HTP) and anaerobic digestion is a promising method to treat these wastes, provided that digestion inhibition is reduced. For this reason, the aim of this work was to investigate the optimal HTP temperature (140–180°C) for biogas production during anaerobic digestion of dead pigs in batch systems. In addition, the effects of hydrochar addition (6 g/L) on anaerobic digestion of pork products after HTP in continuous stirred tank reactors (CSTR) were determined. According to the results, 90% of lipids and 10% of proteins present in the pork were decomposed by HTP. In addition, the highest chemical oxygen demand (COD) concentration in liquid products (LP) reached 192.6 g/L, and it was obtained after 170°C HTP. The biogas potential from the solid residue (SR) and LP was up to 478 mL/g-VS and 398 mL/g-COD, respectively. A temperature of 170°C was suitable for pork HTP, which promoted the practical biogas yield because of the synergistic effect between proteins and lipids. Ammonia inhibition was reduced by the addition of hydrochar to the CSTR during co-digestion of SR and LP, maximum ammonia concentration tolerated by methanogens increased from 2.68 to 3.38 g/L. This improved total biogas yield and degradation rate of substrates, reaching values of 28.62 and 36.06%, respectively. The acetate content in volatile fatty acids (VFA) may be used as an index that reflects the degree of methanogenesis of the system. The results of the present work may also provide guidance for the digestion of feedstock with high protein and lipid content.

## Introduction

China as a country displays an annual production of 451 million swines. For this reason, China is the largest pork producer in the world (National Bureau of Statistics of China [NBSC], 2018). Unfortunately, during the growing process, more than 22 million pigs (5%) die every year of different illnesses, because of inadequate feeding conditions and unadvanced medical technology (Bono et al., 2014). Since 2018, African swine fever (ASF) outbreaks have been a major threat to pig farmers because of an extraordinary variability, a rapid transmissibility and high antibiotics resistance (Blome et al., 2020). As of November 22, 2019, a total of 160 ASF outbreaks had been reported in China, events that caused the culling of 11.93 million hogs (Ding and Wang, 2020). These data emphasizes the importance of timely vaccination, improving the sanitary conditions in piggeries, and reducing the stocking density of pigs. In this context, burial and burning are the usual methods used in China; however, reduced land resources and environmental safety are growing concerns. For this reason, during the last years, the use of biological treatments has been promoted by the government (Wei et al., 2015). Anaerobic digestion of animal carcass for the conversion of these organic wastes into biogas is attracting extensive attention (Russo and von Blottnitz, 2017). Nevertheless, since dead pigs may contain pathogens and some veterinary drugs, a hygienization pretreatment is obligatory before carcass disposal or utilization. According to the regulation published by the Chinese government, livestock carcass should be pretreated at 135°C and 3 bar for 30 min before anaerobic digestion (Ministry of Agriculture and Rural Affairs of The People’s Republic of China [MARF], 2017). This sterilization condition is consistent with that prescribed by the European Community, which indicates conditions of 133°C and 3 bar for 30 min or 140°C and 5 bar for 20 min (Gwyther et al., 2011).

Hydrothermal pretreatment (HTP) has been widely applied before anaerobic digestion of different substrates including food waste, biomass, and municipal sludge (Ding et al., 2017; Li et al., 2017; Ahmad et al., 2018). This technology is also suitable for animal carcass disinfection before anaerobic digestion. Temperature is the dominant HTP factor affecting the decomposition of organic matter. Since organic components are converted into hydrochar at high temperatures, proper HTP temperature for food waste treatment should not exceed 180°C (Munir et al., 2018). Several works about the HTP treatment of slaughterhouse waste (SW) and anaerobic digestion have been published by 133 and 140°C (Eftaxias et al., 2018; Spyridonidis et al., 2018); however, the optimal HTP temperature for biogas production from animal carcass is still not well known. Moreover, the biogas production may be inhibited by ammonia and long chain fatty acids (LCFA), which result from the digestion of protein and lipids (Latifi et al., 2019). The inhibition of biogas production can be reduced by the addition of hydrochar, which contains different functional groups at the surface that promote electron transfer reactions. For example, −OH may increase the pH of the system after combination with H^+^. Also, under alkaline conditions, different groups containing carbon (C≡C, C=O, and C−O) may promote the release of protons (Fagbohungbe et al., 2017). In addition, the porous structure and alkaline environment of hydrochar supports microbial proliferation and enhances buffer capacity, respectively (Choe et al., 2019; Usman et al., 2020).

The objectives of the present study were: (1) investigate the optimal HTP temperature for biogas production during anaerobic digestion of dead pigs; and (2) evaluate the effects of hydrochar addition on anaerobic digestion of pig carcass.

## Materials and Methods

### Feedstock and Inoculum

**FIGURE 1:**
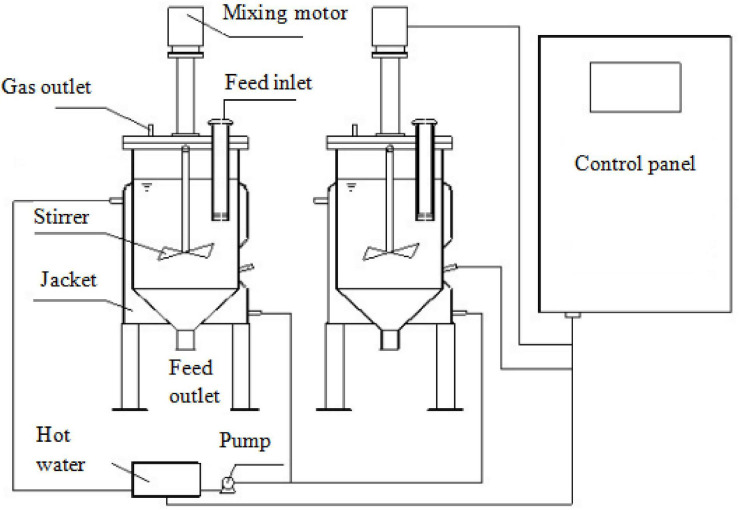
Diagram of the continuous anaerobic digestion system used in the present research.

Because of safety constraints, fresh pork from a market in Hangzhou, China, was used in the investigation as an alternative to dead pigs (Dai et al., 2015). The pork was homogenized using a blender (CPEL-23, Shanghai Guosheng, China) and later stored at −20°C until further use. Rice straw (RS) obtained from a farm in Hangzhoug was used as material for hydrochar preparation. In addition, raw sludge, which was used as inoculum, was collected from a mesophilic biogas plant located in Hangzhou, China. After sampling, the sludge was stored at room temperature (about 25°C) in an airtight container. Physicochemical properties of feedstock and inoculum are shown in Table 1. The functional groups present in raw sludge are shown in Figure 1.

**TABLE 1:** Physicochemical properties of feedstock and inoculum.

	Total solids (TS) (%, w.b.)	Volatile solids (VS)/TS (%, d.b.)	Protein (%, w.b.)	Lipid (%, w.b.)	pH	C/N
Pork	45.9 ± 0.3	97.5 ± 0.1	16.4 ± 0.3	24.6 ± 0.5	6.1 ± 0.1	8.0 ± 0.5
Inoculum	12.9 ± 0.2	52.9 ± 0.2	ND	ND	6.9 ± 0.1	7.0 ± 0.5

*ND, not determined.*

### Hydrothermal Pretreatment

A 2-L stainless reactor (Parr 4848, United States) was used to carry out the HTP and hydrothermal carbonization (HTC) experiments. For HTP, 250 g (wet base, w.b.) pork and 250 mL deionized water were treated at 140, 150, 160, 170, and 180°C for 30 min. For the hydrochar preparation, 100 g RS (dry base, d.b.) and 1,000 mL deionized water were heated at 260°C for 2 h. After cooling to ambient temperature, the solid and liquid final products were separately collected.

### Batch Anaerobic Digestion System

The pork biogas production at different HTP was carried out in batch anaerobic digestion systems, which consisted of a 500-mL glass bottle, a 1-L glass bottle filled with a diluted hydrochloric acid solution (pH < 3), and a 500-mL plastic bottle acting as the bioreactor, the biogas collection bottle, and the liquid collection bottle, respectively.

After HTP, 15 g (w.b.) solid residues (SRs), 25 mL liquid products (LP), and the mixtures of SR (2.81 g-VS, d.b.) and LP [0.22–1.97 g-chemical oxygen demand (COD)] were digested separately. 70 g inoculum (w.b.) was loaded into each bioreactor, and the corresponding ratios of inoculum to substrate (I/S) are displayed in Table 2 (Xu et al., 2017). After loading with feedstock and inoculum, the working volume of the bioreactor was adjusted to 150 mL by adding deionized water and the top space was flushed with N_2_ for 5 min. The bioreactors were kept at 35 ± 1°C on a water bath and manually shaken for 1 min twice a day. All the digestions were run in duplicate for 26 days until no biogas production was observed over a 5-day period.

**TABLE 2:** The results for biogas production during batch digestion of pork products after HTP.

		140°C	150°C	160°C	170°C	180°C
I/S ratio	SR	1.30 ± 0.1	1.40 ± 0.1	1.53 ± 0.1	1.71 ± 0.1	1.95 ± 0.1
	LP	1.92 ± 0.1	1.58 ± 0.1	1.27 ± 0.1	0.99 ± 0.1	1.50 ± 0.1
	SR + LP	1.83 ± 0.1	1.50 ± 0.1	1.30 ± 0.1	1.00 ± 0.1	1.40 ± 0.1
Biogas yield (mL/g-VS)	SR	263.2 ± 13.9	289.3 ± 15.2	352.1 ± 15.1	422.2 ± 16.6	479.3 ± 18.9
	LP	398.1 ± 11.4	312.2 ± 13.3	235.3 ± 12.6	174.2 ± 11.2	291.4 ± 10.7
	SR + LP	392.4 ± 16.2	326.4 ± 15.6	272.4 ± 12.8	211.3 ± 14.4	315.3 ± 14.3
Methane content (%)	SR	69.4 ± 0.6	71.2 ± 0.6	71.8 ± 0.5	72.2 ± 0.5	73.3 ± 0.6
	LP	73.4 ± 0.6	71.8 ± 0.5	70.3 ± 0.6	69.7 ± 0.6	69.1 ± 0.8
	SR + LP	72.6 ± 0.4	71.2 ± 0.4	71.0 ± 0.5	70.3 ± 0.6	69.3 ± 0.7
TAN (g/L)	SR	2.06 ± 0.05	1.94 ± 0.04	1.75 ± 0.03	1.56 ± 0.02	1.42 ± 0.02
	LP	0.39 ± 0.02	0.51 ± 0.02	0.72 ± 0.03	0.89 ± 0.04	1.03 ± 0.05
	SR + LP	1.86 ± 0.03	2.01 ± 0.03	2.12 ± 0.03	2.27 ± 0.03	2.08 ± 0.03
pH	SR	7.49 ± 0.06	7.35 ± 0.06	7.23 ± 0.05	7.14 ± 0.08	7.03 ± 0.07
	LP	7.01 ± 0.08	6.85 ± 0.08	6.73 ± 0.07	6.64 ± 0.06	6.52 ± 0.07
	SR + LP	7.21 ± 0.06	7.15 ± 0.06	7.13 ± 0.07	7.08 ± 0.07	7.02 ± 0.08
Theoretical biogas yield (L)	SR	11.77 ± 0.46	10.48 ± 0.46	8.98 ± 0.46	6.87 ± 0.46	4.29 ± 0.46
	LP	3.97 ± 0.09	4.82 ± 0.09	5.97 ± 0.09	7.67 ± 0.09	5.09 ± 0.09
	SR + LP	15.74 ± 0.55	15.3 ± 0.55	14.95 ± 0.55	14.54 ± 0.55	9.38 ± 0.55

*I/S ratio, the ratio of inoculum to substrate.*

### Anaerobic Digestion With Continuous Stirred Tank Reactors

**FIGURE 2:**
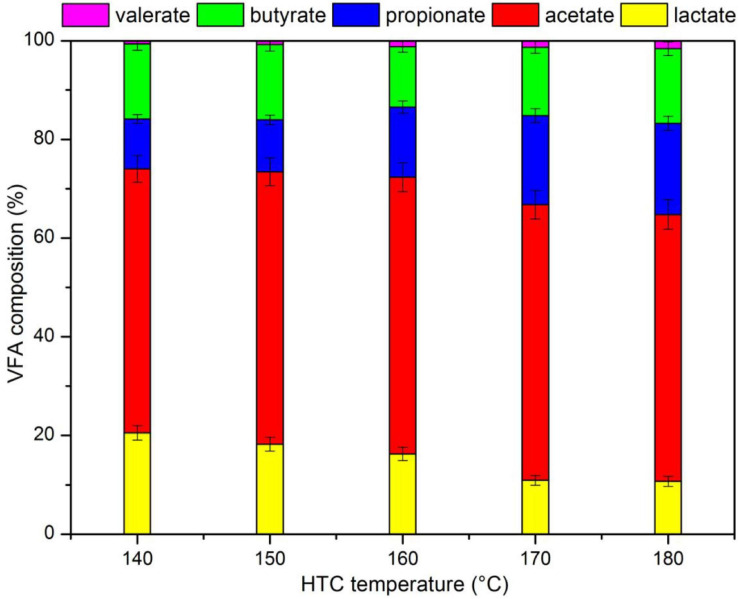
Volatile fatty acids (VFA) composition of liquid products (LP) after hydrothermal pretreatment (HTP).

After completing the batch digestion, the optimal HTP temperature (170°C) was determined. Based on this temperature, the pretreated pork products were digested in continuous stirred tank reactors (CSTR). The schematic diagram of CSTR is displayed in Figure 2. The CTSR consisted of two reactors (30 L) and a central control. The reactors were set to operate at 37 ± 1°C and filled with 20 L inoculum before start-up.

Two sets of digestions were carried out. In order to determine the standard deviation of the biogas yield from the two reactors, the co-digestion of SR and LP were performed in duplicate for 20 days. After HTP of 250 g pork, the resulting products (35.85 g-VS SR and 48.15 g-COD LP) were added to each reactor on a daily basis. The amounts were equivalent to an organic loading rate (OLR) of 4.2 g-VS⋅(L⋅d)^–1^. In addition, the reactors were stirred for 20 min at 35 rpm every 2 h, and the biogas production was measured using a drumtype gas flowmeter (Alpha LML-2, Changchun, China).

After data was collected to determine the standard deviation, the reactors were restarted with the same operational parameters. According to data reported in previous studies, hydrochar addition of 2–10 g/L resulted in 60.7–90.8% increase in biogas production from SR. This occurred because hydrochar increased the presence of functional groups as well as the buffer capacity of the system (Xu et al., 2018). In the present research, 6 g/L hydrochar were added to one reactor (A) to reduce process inhibition; in addition, another reactor (B), with no hydrochar addition, was set as the control group. During digestion and according to biogas production performance, the reactor was manually fed and discharged once a day between days 1 and 10, once every 2 days between days 12 and 18, and every 3 days between days 21 and 27. Digestate was collected from each reactor and subsequently analyzed.

### Analytical Methods

Total solids (TS), volatile solids (VS), total ammonia nitrogen (TAN), and COD were measured according to the APHA (2006) standard method. The pH value was determined using a pH meter (PHS-3D, Shanghai, China). Total carbon and nitrogen content were measured with an elemental analyzer (EA 1112, CarloErba, Italy). Protein content was determined with a Kjeldahl nitrogen determination device (UDK152, Italy; protein = 6.25 × total nitrogen). For this purpose, the dry pork was digested with concentrated sulfuric acid at 420°C for 90 min; later, the solution was distilled with a sodium hydroxide and boric acid solution for 5 min. Finally, a titration with hydrochloric acid solution was performed. Lipid content was determined after Soxhlet extraction (SXT-06, Shanghai). Herein, the fat present in the pork was extracted using petroleum ether. Heating was provided with a water bath (80°C) for 4 h. Later, petroleum ether was evaporated and sample dried at 120°C. The composition of the biogas and volatile fatty acids (VFA) produced during the digestion were measured using gas chromatography (GC 2014, Shimadzu, Japan). In order to determine biogas composition, 10 μL gas were analyzed with a thermal conductivity detector. The temperatures of the column, injector port, and detector were 100, 120, and 120°C, respectively. The carrier gas was argon and was supplied at a flow rate of 30 mL/min. For VFA analysis, the fermentation liquid was centrifuged at 10,000 rpm for 15 min, acidified with metaphosphoric acid until reaching a pH value < 2.5, and filtered through a 0.22 μm membrane. After pretreatment, 0.4 μL of sample were analyzed using a hydrogen flame ionization detector. The temperatures of the injector port and detector were 250 and 280°C, respectively. The carrier gas was argon and was supplied at a flow rate of 30 mL/min. The functional groups present on the hydrochar and sludge were characterized at room temperature using Fourier transform infrared (FTIR) spectroscopy (Varian 640-IR, United States). For this purpose, the range of 400–4,000 cm^–1^ and KBr method were selected. Hydrochar surface area, pore volume, and pore size were measured with the Brunauer–Emmet–Teller (BET) method using an automatic nitrogen adsorption analyzer (JW-BK, China).

## Results and Discussion

### Effects of Hydrothermal Temperature on Degradability and Biogas Production of Pork Products

#### Degradability of Pork Products After HTP

Characteristics of pork products after different HTP temperatures are shown in Table 3. According to the results, during HTP, the organics present in the pork presented a 50% reduction. Almost 90% of lipids were decomposed at 140°C, while at 180°C more than 85% of the protein was still preserved in the SR. The results were consistent with other study that indicated that most lipids were dissolved at 160°C and the soluble protein content increased as hydrothermal temperature increased (Pavlovič et al., 2013). This occurred because above 160°C, the cell walls are ruptured liberating the protein present inside the cell (Zhang et al., 2014). The COD concentrations in LP were 99.8–192.6 g/L after HTP, and the VFA content in LP was between 15.2 and 25.6 g/L. VFA only contributed to 13.2–19.1% of total COD, because the preliminary decomposition during HTP resulted in proteins and lipids hydrolization to produce polypeptides and LCFA, respectively. For example, a COD concentration of 149.6 g/L was achieved after SW was subjected to a HTP of 140°C. In addition, VFA accounted for less than 10% of total COD (Spyridonidis et al., 2018). Although the breakdown of organic compounds present in the biomass was accelerated at increasing HTP temperatures, the maximum values of COD and VFA were obtained after 170°C HTP. It is likely that some micromolecular organics such as VFA were converted into gas when temperature increased from 170 to 180°C. It has been reported that during HTP, soluble organics with molecular weight <10 kDa were easily converted into gas or refractory materials (Liu et al., 2012).

**TABLE 3:** Characterization of solid residue (SR) and liquid products (LP) after different hydrothermal pretreatment (HTP) temperatures.

	140°C	150°C	160°C	170°C	180°C
TS (%, w.b.)	25.0 ± 0.2	23.3 ± 0.2	21.2 ± 0.3	19.0 ± 0.3	16.6 ± 0.4
VS/TS (%, d.b.)	98.3 ± 0.1	98.1 ± 0.2	98.7 ± 0.2	98.2 ± 0.1	98.5 ± 0.2
Lipid (%, w.b.)	3.3 ± 0.1	2.7 ± 0.1	2.4 ± 0.1	2.2 ± 0.1	2.1 ± 0.1
Protein (%, w.b.)	21.5 ± 0.3	20.1 ± 0.3	18.5 ± 0.3	16.4 ± 0.3	14.1 ± 0.3
Chemical oxygen demand (COD, g/L)	99.8 ± 0.3	121.2 ± 0.3	150.0 ± 0.3	192.6 ± 0.3	127.8 ± 0.3
Ammonia (g/L)	2.0 ± 0.2	3.8 ± 0.2	4.3 ± 0. 2	6.5 ± 0.2	4.9 ± 0.1
pH	6.32 ± 0.1	6.28 ± 0.1	6.25 ± 0.1	6.21 ± 0.1	6.19 ± 0.1
Volatile fatty acids (VFA, g/L)	15.2 ± 0.2	18.6 ± 0.2	21.2 ± 0.2	25.6 ± 0.2	20.6 ± 0.2

**FIGURE 3:**
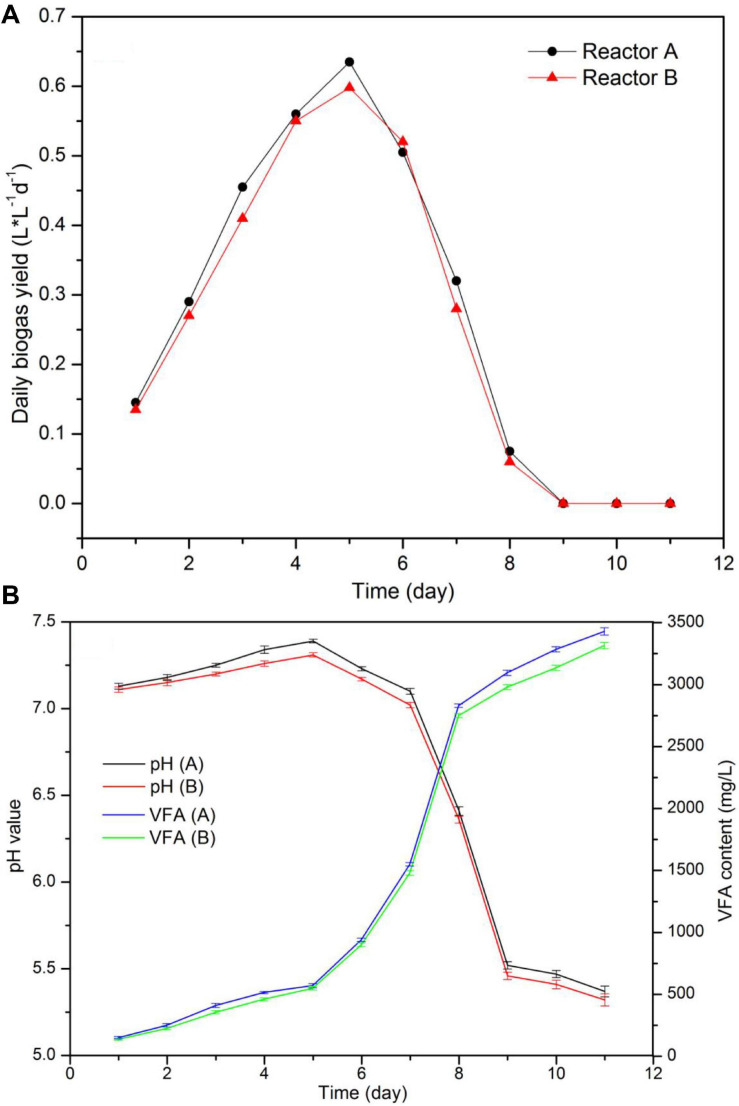
Performance of continuous stirred tank reactors (CSTR) digestion of pork products after HTP: **(A)** daily biogas production; **(B)** pH value and VFA content.

Figure 3 shows the VFA composition in the LP. Acetate accounted for about 50% of VFA, followed by lactate or propionate (10–20%), butyrate (10%), and valerate (1%). Part of the lactate was converted into propionate with increasing HTP temperatures. It was likely that propionate could be transformed from H_2_ and lactate (Grause et al., 2012), and the hydrogen content in biomass was reduced with increasing temperatures during HTP (Yan et al., 2018); the increase in HTP temperature promoted the formation of H_2_ and the conversion of lactate to propionate due to subcritical condition. The possible reaction for the conversion of lactate to propionate is displayed in Table 4.

**TABLE 4:** Gibbs’s energy of lactate converted to acetate, propionate and butyrate.

Equilibrium	ΔG° (kJ/mol)
Lactate + 2H_2_O → acetate + HCO_3_^–^ + H^+^ + 2H_2_	−4.2
Lactate + H_2_ → propionate + H_2_O	−79.9
2Lactate + H^+^ → butyrate + 2H_2_ + 2CO_2_	−64.1

#### Specific Biogas Yield of Pork Products After HTP

Table 2 displays the results for biogas production during batch digestion of pork products after HTP. The specific biogas yield from SR and from LP reached 479 mL/g-VS and 398 mL/g-COD at the I/S ratio of 1.95 and 1.92, respectively; both biogas yields decreased with reducing I/S ratio. The methane content in the biogas was between 69.1 and 73.4% during the whole digestion process, value that agree with the high theoretical methane content coefficient for lipids and proteins (Wu et al., 2009). Although the theoretical biogas potential of SW is above 740 mL/g-VS, because of the inhibition by excess ammonia and LCFA, the specific biogas yield normally depends on the operational conditions (Wu et al., 2015). Other studies have reported SW methane yields between 300 and 800 mL/g-VS. For example, a biogas yield of 443 mL/g-VS was obtained during the digestion of SW at a I/S ratio of 4 (Latifi et al., 2019). Also, a biogas yield of 425 mL/g-COD was obtained after SW water digestion with an OLR of 1.82 g⋅(L d)^–1^ (Schmidt et al., 2018). These results are close to biogas yields obtained in this paper.

A TAN concentration of 1.7 g/L was considered as the threshold for ammonia inhibition during the digestion of substrates with high nitrogen content (Akindele and Sartaj, 2018). In the present experiments, the biogas yield decreased from 422 to 289 mL/g-VS when the TAN value increased from 1.53 to 1.94 g/L. This occurred because the protein content of the substrate increased from 16.4 to 20.1%. During the co-digestion of SR and LP and at the same I/S ratio, the biogas yield was higher than that obtained during mono-digestion of either SR or LP. Nevertheless, during co-digestion, the TAN value was higher. This has been attributed to the increasing buffer capacity resulting from the synergistic effect between VFA and ammonia (Zhang et al., 2013). Eq. 1 displays the formation of the buffer system.

(1) $${\text{C}}_{x}\,{\text{H}}_{y}\,\text{COOH}+{\text{NH}}_{3}\cdot {\text{H}}_{2}\,\text{O}\to {\text{C}}_{x}\,{\text{H}}_{y}\,{\text{COO}}^{-}+{\text{NH}}_{4}^{+}+{\text{H}}_{2}\,\text{O}$$

The calculation of the theoretical total biogas yield from pork products after HTP was based on the specific SR and LP biogas yield (479 mL/g-VS and 398 mL/g-COD, respectively). Results are shown in Table 2. According to the theoretical results, the highest biogas yield from pork products would be obtained after 140°C HTP. However, the risk of inhibition due to ammonium was present. It was also determined that, because of the synergistic effect between protein and lipid, a temperature of 170°C was the optimal value for pork HTP, which would promote the maximum value for practical biogas yield.

### The Parallel CSTR Digestion of Pork Products After HTP

**FIGURE 4:**
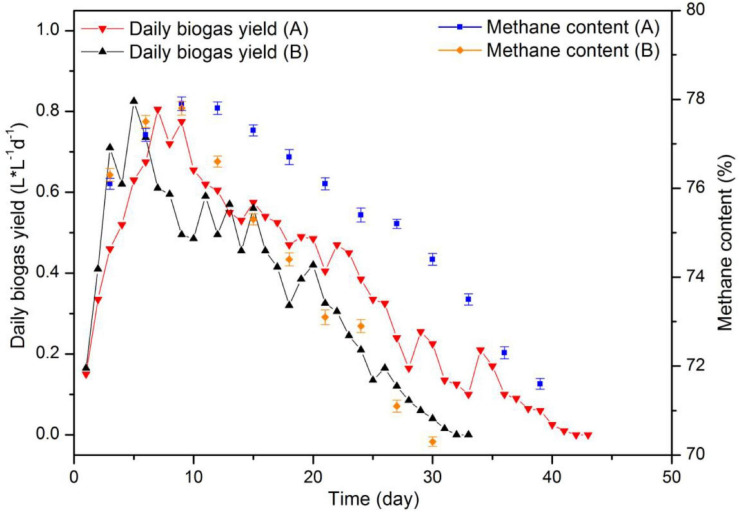
Biogas yield and methane content in CSTR digestion of pork products after HTP.

The daily biogas production and process stability from the two reactors running at the same conditions is shown in Figure 4. As data indicated, the biogas production increased during the first 5 days from 0.14 L⋅L^–1^⋅d^–1^ to around 0.61 L L^–1^ d^–1^, and it was rapidly reduced after day 6 and stopped after day 9. Moreover, VFA content augmented from around 920–3400 mg/L between days 6 and 9. According to pH values and VFA content, the reduction in biogas production was the result of the decrease in pH and VFA accumulation, a process that was caused by the addition of sour LP because of improper storage. It is known that the addition of sour substrate with pH values between 5.0 and 5.5 may cause digester instability. As a consequence, a rapid acidification and reaction failure may easily happen (Kong et al., 2016). When food waste was soaked and stored with no refrigeration for 12 h, pH decreased from 6.5–7.4 to 5.2–5.5, causing a rapid increase from 0.03 to 0.4 in the VFA/COD value (Kuruti et al., 2017).

Although the biogas production only occurred during the first 8 days of the process, the values for daily biogas yield, pH, and VFA content in the two reactors were very close during this period. Total biogas production reached 59.7 and 56.46 L in each reactor, the difference in biogas production between the two reactors was less than 7%.

### Continuous Stirred Tank Reactors Digestion of Pork Products After HTP With Hydrochar Addition

#### Impact of Hydrochar Addition on Biogas Production

**FIGURE 5:**
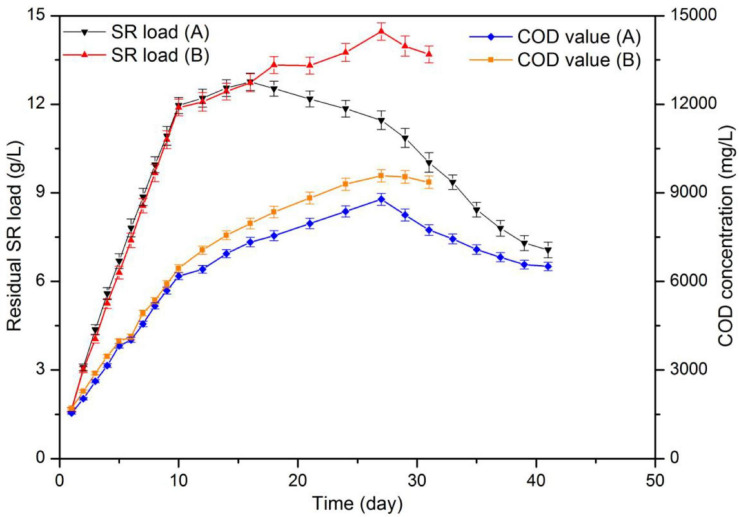
Substrate load and chemical oxygen demand (COD) values during CSTR digestion of pork products after HTP.

**FIGURE 6:**
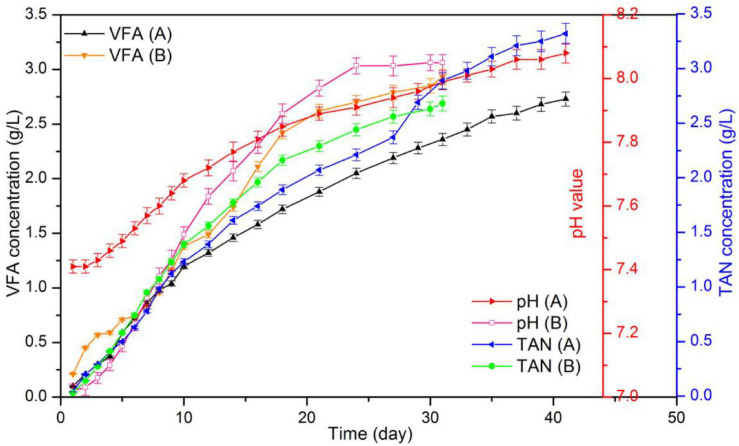
pH and total ammonia nitrogen (TAN) concentration in VFA during CSTR digestion of pork products after HTP.

Figure 5 displays the results for biogas yield and Figure 6 those for residual SR load and COD concentration of fermentation liquor. Both reactors displayed a rapid increase in daily biogas yield during the first 9 days of digestion. In addition, with an OLR of 4.2 g-VS (L d)^–1^, the substrate load increased to 10.8 g/L. Maximum daily biogas yield values were 0.820 and 0.805 L L^–1^d^–1^ for each reactor. A drop on biogas production occurred in the two reactors after day 9, maybe as a result of the high protein content in SR, indicating that the reactors were overloaded. Therefore, during digestion, the two reactors were fed on days 1–10, 12, 14, 16, 18, 21, 24, and 27. The corresponding OLR were 2.1 g-VS (L d)^–1^ between days 11–17 and 1.4 g-VS (L d)^–1^ between days 18 and 30. Prolonging feeding periods and decreasing OLR are common remediation strategies when better degradation rates of the accumulated substrate are intended. It has been reported that a decline in biogas production and an accumulation in VFA were observed at the OLR of 2.3 g (L d)^–1^ during digestion of SW. Also, the OLR decreased to 1.5 g (L d)^–1^ after a starvation period of 10 days (Eftaxias et al., 2018).

After day 9, both reactors showed fluctuations in daily biogas yield. The reason is that the added volume and COD amount of substrate in both reactors immediately increased and created a temporary inhibition. At day 22 and when the substrate load was up to 13.3 g/L, the daily biogas production was less than half that of the maximum value. Biogas production ended at day 31 and day 41, respectively. The total biogas yield of 309.2 L was achieved in the reactor with hydrochar addition, which represents a 28.62% higher than that (240.4 L) in control group.

In addition, the reactor with hydrochar addition displayed methane content between 71.6 and 77.9%, and that for control reactor was between 70.3 and 77.8%. The methane content first increased and later decreased as a consequence of a declined biogas production. Nearly 30% of CH_4_ resulted from the reaction between CO_2_ and H_2_ (Yuan and Zhu, 2016).

After digestion, the control group showed a residual SR load and COD concentration of 13.69 and 9.36 g/L, respectively. In addition, the values in the reactor containing hydrochar were 7.07 and 6.51 g/L, correspondingly. Substrates containing 609.45 g-VS SR and 818.55 g-COD LP were added into each reactor during digestion. According to changes in volume load and COD, it was calculated that 294.52 g-VS SR and 414.55g-COD LP were decomposed in the control group during biogas production. On the other hand, the amounts were 406.59 g-VS SR and 558.15 g-COD LP in another reactor. The average specific biogas yields were 339.0 and 320.5 mL-g/VS in control and treatment, respectively. In the control reactor, the VS removal rates for SR and LP were 48.33 and 50.64%, respectively. After hydrochar addition, these values increased to 66.77 and 68.19%, respectively. In both reactors, SR removal rates were similar to those of LP. These results suggested that the degradation of proteins and lipids may occur in a synchronous way. In theory, the hydrolysis of lipids was slower than that of proteins. The results suggested that the hydrolysis of lipids was promoted by HTP, which was able to increase the buffer capacity of the system through the synergistic effect of proteins and lipids. Moreover, at a inoculum/decomposed substrates ratio of 1.41, the specific biogas yield was 320.5 mL-g/VS. This value is similar to a yield of 315.3 mL-g/VS, which was obtained during the co-digestion of SR and LP at a I/S ratio of 1.40. Although with high theoretical biogas potential, the practical biogas yield from digestion of SW depends on operational parameter, such as the characteristics of the feedstock and inoculum, OLR and pretreatment method (Hu et al., 2018). The performance of mesothermal semi-continous digestion of SW in other studies were shown in Table 5, and the high methane yield of SW was obtained with a low OLR; it suggested that the low methane yield in this study was due to the overload of substrate.

**TABLE 5:** Performance of mesothermal semi-continous digestion of slaughterhouse waste (SW) in recent literatures.

**Thermal treatment**	Working volume (L)	Methane Yield (L/g-VS)	Organic loading rate [OLR, g⋅(L d)^–^^1^]	Substrate	References
70°C, 2 h	8.0	0.640	1.3	SW	Escudero et al., 2014
Not treated	6.0	0.350	2.5–3.5	N-rich SW	Ortner et al., 2014
Not treated	11.0	0.291	1.1	lipid-rich SW	Rodríguez-Méndez et al., 2017
133°C, 3 bar, 20 min	42.0	0.408	1.5–10	SW + Ni, Co, Mo	Eftaxias et al., 2018
121°C, 30 min	1.8	0.588	0.85–1.00	High-fat SW	Harris et al., 2018
Not treated	14.8	0.574	NM	SW + sludge	Latifi et al., 2019

*SW, slaughterhouse waste; NM, not mentioned.*

In addition, the net energy was calculated by subtracting the energy consumed in HTC from the methane energy produced during hydrochar addition. The increase in methane energy (kJ) was evaluated by multiplying the lower heating value of methane (35.89 kJ/L) by the increased methane yield (L). Energy consumption for HTC was analyzed based on Mustafa et al. (2018) who reported a value of 1,092 kJ/kg at 260°C. It was determined that, after hydrochar addition, methane yield increased by 48.2 L which in total represents an energy yield of 1,728 kJ. It was also determined that 2,402 kJ were consumed during HTC of 200 g biomass and 2 L water. Even in the present experiments HTC displaced a high-energy consumption, a positive value for net energy can be obtained if the biogas inhibition is further ameliorated by hydrochar addition.

#### Process Stability

**FIGURE 7:**
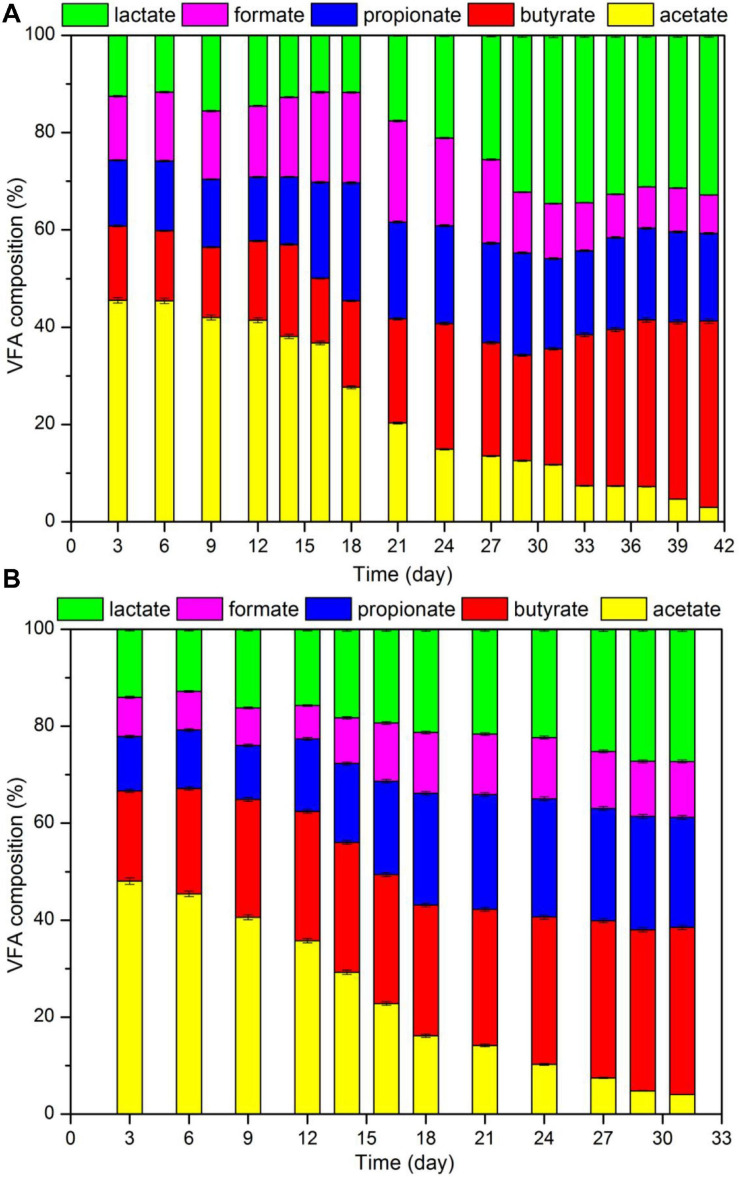
Composition of VFA in the fermentation liquid during CSTR digestion of pork products after HTP: **(A)** reactor with hydrochar addition; **(B)** control group.

**FIGURE 8:**
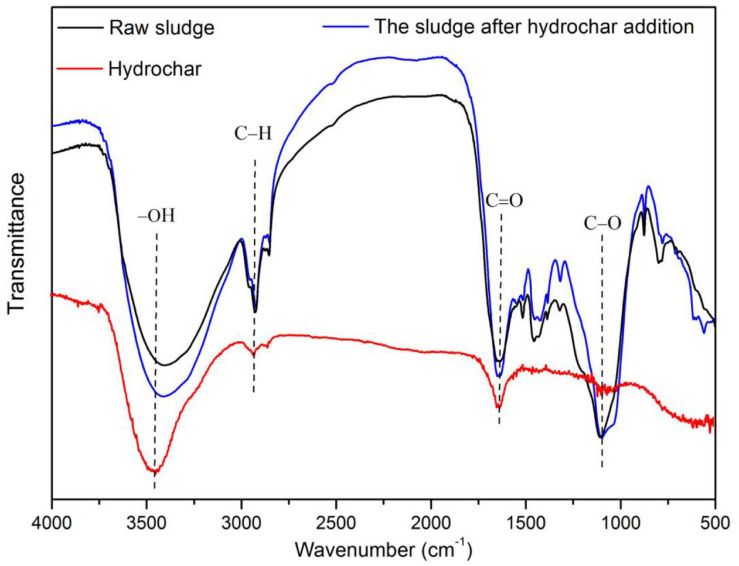
The changes on the functional groups of inoculated sludge after hydrochar addition.

Figure 7 displays the results for pH, TAN and VFA during the digestion process. According to the data, in the control group the initial and final pH were 7.02 and 8.05, respectively. In addition, the final TAN and VFA concentrations were 2.68 and 2.93 g/L, respectively. Data also indicated that in the hydrochar treated reactor, pH increased from 7.41 to 8.08, and the concentrations of TAN and VFA were 3.38 and 2.73 g/L, respectively. Usually, ammonia and LCFA hinder the digestion of substrates with high protein and lipid content. High ammonia concentrations usually cause that the digesters operate within a neutral pH range, which is known as “inhibited steady state.” This state results in a low biogas yield, VFA accumulation, and a drop in pH values (Shi et al., 2017). LCFA are readily adsorbed on the cell membrane, limiting the mass transfer between methanogens and substrate. LCFA inhibition also occurs along with acetate accumulation as hydrolysis product (Harris et al., 2018). Figure 8 shows VFA composition. According to the data, no accumulation of acetate were observed in the reactors. Moreover, at day 16, TAN concentrations in both reactors occurred above the inhibition threshold of 1.7 g/L. These results may indicate that the decrease in biogas production occurred because of high ammonia concentrations.

Data also indicated a significant and positive relationship between acetate content and biogas production. At the beginning of the digestion process, the acetate content was more than 40%. When acetate content was decreased to 20%, daily biogas yield was reduced to half that of the peak value; in addition, methanogenesis nearly stopped when acetate content was below 5%. Since 72% methane was produced during acetate degradation, acetate was almost totally spent. On the other hand, during digestion, lactate, propionate, and butyrate accumulated (Yuan and Zhu, 2016). Thus, acetate content in VFA may be used as the index that reflects the degree of methanogenesis of a system.

#### The Role of Hydrochar on Improving Biogas Production

The increase of 28.62% in biogas yield was achieved when hydrochar was added in concentrations that reduced ammonium inhibition. In this case, ammonia tolerance increased from 2.68 to 3.38 g/L. Inhibition of ammonia on methanogens is mainly due to free ammonia (FA), since FA is able to permeate the cell membrane and limit electron transfer (Fajardo et al., 2014).

It has been reported that hydrochar addition enhances biogas production through different mechanisms. For example, hydrochar promotes electron transfer reactions because of the presence of surface functional groups, improves the buffer capacity of the system by providing an alkaline environment, and supports microbial proliferation through the porous structure (Fagbohungbe et al., 2017; Usman et al., 2020). Previous reports have indicated that biogas yield increased in 64 and 52% by hydrochar addition during the digestion of fish processing waste and hydrothermal liquefaction wastewater, respectively (Choe et al., 2019; Usman et al., 2020). As shown in Figure 1, the functional groups (–OH and C=O) present in the inoculated sludge increased after hydrochar addition. The porous properties of the hydrochar used in this study are presented in Table 6. According to the data, the porous structure of hydrochar, with the BET surface area of 19.4 m^2^/g, was also beneficial for methanogens growth.

**TABLE 6:** Pore properties of hydrochar.

	Value
BET surface area (m^2^/g)	19.4 ± 0.1
Total pore volume (cm^3^/g)	0.063 ± 0.002
Average pore size (nm)	13.0 ± 0.1

## Conclusion and Perspective

This study demonstrated the feasibility of anaerobic digestion of pork products after HTP. According to the HTP results, most lipids broke down at 140°C, and protein decomposition was accelerated at temperatures above 160°C. The biogas yield of 320 mL/g-VS was achieved during CSTR digestion of pork products after HTP. It suggests that the ORL should not exceed 4.2 g-VS (L d)^–1^ to prevent inhibition when pork waste was fed as substrate, a value similar to the case of food waste. Hydrochar addition offered a good option by reducing the ammonia inhibition. In the present work, hydrochar was added to the reactor only one time. However, hydrochar was gradually lost with the discharge of digestate. As remedy, the recycling of the digested effluents and continuous supplement of hydrochar to the reactors may be an option. Thus, a study about the optimal parameters and conditions for hydrochar addition during the continuous digestion process should be considered.

The results presented herein provide an option for a harmless utilization and treatment of animal carcass. Because of the advantage of biogas recovery, anaerobic digestion is the mainstream treatment for food waste in China. Since the increasing demand in environment protection and clean energy, the treatment of animal carcass by HTP and anaerobic digestion are encouraged. Assuming a centralized collection and sterilization for animal carcass, its co-digestion with hydrochar in large-scale digesters will be a promising way for the harmless treatment method.

## Data Availability Statement

The original contributions presented in the study are included in the article/supplementary material, further inquiries can be directed to the corresponding author.

## Author Contributions

All authors contributed equally to the article and approved the submitted version.

## Conflict of Interest

The authors declare that the research was conducted in the absence of any commercial or financial relationships that could be construed as a potential conflict of interest.
